# Spatial cognition and the avian hippocampus: Research in domestic chicks

**DOI:** 10.3389/fpsyg.2022.1005726

**Published:** 2022-09-23

**Authors:** Anastasia Morandi-Raikova, Uwe Mayer

**Affiliations:** Center for Mind/Brain Sciences, University of Trento, Rovereto, Italy

**Keywords:** avian hippocampus, spatial memory, navigation, place cells, immediate early genes (IEGs), c-Fos

## Abstract

In this review, we discuss the functional equivalence of the avian and mammalian hippocampus, based mostly on our own research in domestic chicks, which provide an important developmental model (most research on spatial cognition in other birds relies on adult animals). In birds, like in mammals, the hippocampus plays a central role in processing spatial information. However, the structure of this homolog area shows remarkable differences between birds and mammals. To understand the evolutionary origin of the neural mechanisms for spatial navigation, it is important to test how far theories developed for the mammalian hippocampus can also be applied to the avian hippocampal formation. To address this issue, we present a brief overview of studies carried out in domestic chicks, investigating the direct involvement of chicks’ hippocampus homolog in spatial navigation.

## Introduction

Domestic chickens are ground-living animals. Thanks to their precocial development young chicks provided an important developmental model for the study of many cognitive functions (Rosa-Salva et al., 2021). Chicks have been also widely used for the study of spatial orientation, complementing the research on spatial cognition in other birds, which almost exclusively used adult animals. Chicks can locate a goal using local cues as a beacon (Regolin et al., 1995), egocentric information (Vallortigara, 1996), distances (Vallortigara and Zanforlin, 1986; Chiesa et al., 2006), and topographical features of the environment (Rashid and Andrew, 1989). They can orient in relation to free-standing objects (Morandi-Raikova et al., 2020) and find the center of a square-shaped enclosure in relation to the walls (Tommasi et al., 1997; Tommasi and Vallortigara, 2000). Chicks can use view matching strategies (“snap-shot memories”) of the visual scenes for navigation (Dawkins and Woodington, 2000; Pecchia and Vallortigara, 2010a; Pecchia et al., 2011; but see Lee et al., 2012) and can also orient by the shape (“geometry”) of a rectangular enclosure (Vallortigara et al., 1990; Chiandetti et al., 2007, 2015; Mayer et al., 2016). In a small scale geometrical orientation task, they can use both the extended surfaces of a rectangular enclosure and a rectangular array of discrete objects (Pecchia and Vallortigara, 2010b, 2012).

Based on behavioral evidence, inferences have been often made on the involvement of chicks’ hippocampus for navigation. However, until recently, only one study directly investigated the role of chicks’ hippocampus in spatial tasks (Tommasi et al., 2003). We have thus performed a series of experiments using neural activity markers (the immediate early gene product c-Fos) to systematically investigate the involvement of chick hippocampus in different spatial tasks (Mayer et al., 2016, 2018; Morandi-Raikova and Mayer, 2020, 2021, 2022). Immediate early genes are expressed in response to neural activation and their products are often used to map neural activation in mammals and in birds (Lanahan and Worley, 1998; Tischmeyer and Grimm, 1999; Smulders and DeVoogd, 2000; Kubik et al., 2007; Golüke et al., 2019; Corrales Parada et al., 2021). Overall, our studies showed remarkable functional similarities in spatial processing between chicks’ hippocampus compared to adults of other bird species and to mammals. In this paper, we will provide a brief overview of the current state of knowledge on the hippocampal structure in birds. We will then present an overview of the studies that directly investigated the involvement of chicks’ hippocampus in spatial orientation, hippocampal lateralization of spatial functions and the neural mechanisms behind the encoding of locations in chicks and other birds.

## Do chicks have a hippocampus?

**Figure 1:**
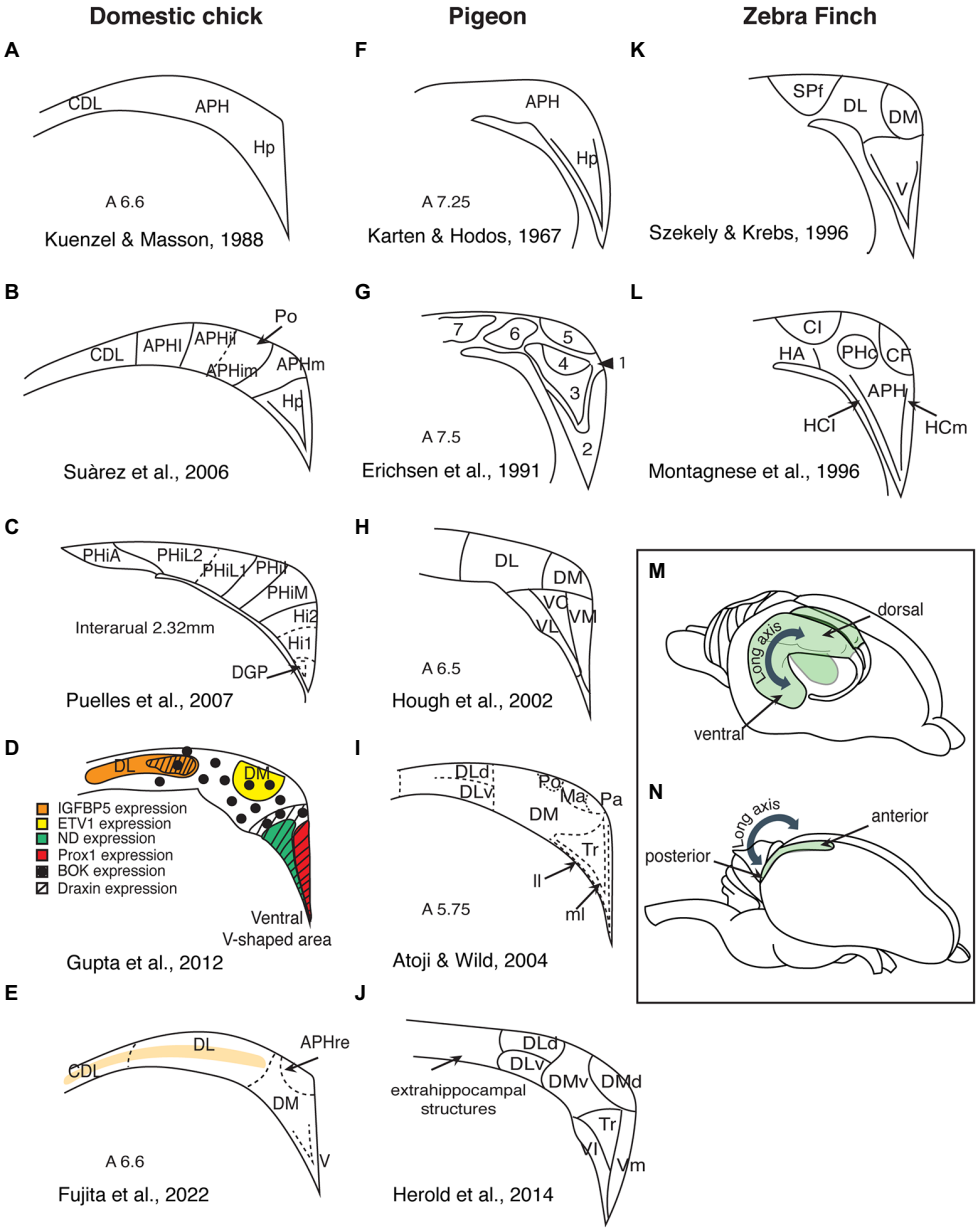
Summary of the different subdivisions proposed for birds hippocampal formation (schematic redrawing). **(A–E)** Domestic chicks; **(F–J)** Pigeons; **(K,L)** Zebra finches; **(M)** Side view of a rodent brain, showing the hippocampal organization along the dorsoventral axis (depicted in green). **(N)** Side view of an avian brain, showing the hippocampal organization along the anteroposterior axis. Hp, hippocampus; CDL, area corticoidae dorsolateralis; DM, dorsomedial; DL, dorsolateral; DLd, dorsal portion of the dorsolateral hippocampal formation; DLv, ventral portion of the dorsolateral hippocampal formation; V, ventral; VL, ventrolateral; VC, ventrocentral; VM, ventromedial; Vl, ventrolateral; Vm, ventromedial; APH, parahippocampal area; APHre, ectopic part of the rostral area parahippocampalis; APHl, lateral parahippocampal area; APHil, lateral part of the intermediate parahipocampal area; APHim, medial part of the intermediate; APHm, medial parahippocampal area; Po, cell-poor region of the hippocampal formation; PHiA, parahipp area, apical; PHiL2, parahipp area, lat 2; PHiL1, parahipp area, lat 1; PHiI, parahipp area, intermed; PHiM, parahipp area, medial; Hi1, hippocampus proper part 1; Hi2, hippocampus proper part 2; DGP, dentate gyrus primordium; Tr, triangular region of the V-shaped hippocampal layer; ll, lateral layer of the V-shaped hippocampal layer; ml, medial layer of the V-shaped hippocampal layer; Ma, magnocellular region of the hippocampal formation; Pa, parvocellular region of the hippocampal formation; SPf, Substance P-immunoreactive nucleus; CL, intermediate corticoid area; HA, apical hyperpallium; PHc, central field of the parahippocampus; *CF*, crescent field; HCl, lateral hippocampus; and HCm, medial hippocampaus.

Although this issue was debated for a long time, it is now widely accepted that the mammalian and the avian hippocampus are homologous structures (Striedter, 2015). The position and anatomical structure of hippocampus varies across and within different taxonomic groups. For instance, the hippocampus of rodents lies within caudal pole of the telencephalon. In primates, however, the hippocampus lies deep within the medial temporal lobe. This “dislocation” within different mammalian species was probably caused by the expansion of the cortex during evolution. Indeed, ontogenetically the mammalian hippocampus develops in the telencephalon’s dorsomedial sector and remains at that location in both marsupials and monotremes, the two “most primitive” mammalian lineages (Hevner, 2016). This is where subsequent research located the hippocampus in all sauropsids (sphenodon, lizards, snakes, turtles, crocodilians, and birds), amphibians, and cartilaginous fishes. For birds, based on topological and functional comparisons, it has been further suggested that the organization of the avian hippocampus along the anterior–posterior axis might be equivalent to the organization along the dorsoventral axis in mammalian hippocampus (Smulders, 2017; Payne et al., 2021, see Figures 1M,N). Among vertebrates, the only exception is the ray-finned fishes (actinopterygians). Due to divergent neural development, their hippocampal homolog occupies the dorsolateral telencephalon (Rodríguez et al., 2006; Moreno and González, 2007; Mueller and Wullimann, 2009). The connections of the avian hippocampus to brain areas like septum, hypothalamus, brainstem nuclei, and sensory processing areas are also similar to those found in other vertebrates, but not fully identical (Casini et al., 1986; Atoji and Wild, 2006; Striedter, 2015).

In rodents, the hippocampus shows a trilaminar organization and can be histologically divided into five subregions (the dentate gyrus and the cornu ammonis regions CA1–CA4). The principal layer of the three layers of the CA regions comprises densely packed pyramidal cells, whereas the dentate gyrus is a separate structure, also consisting of three layers with distinct neurons (molecular, granular, and polymorphic). In lizards too, the hippocampus shows a clear lamination, which is less prominent in turtles and crocodiles (the taxon closest to birds; Striedter, 2015). In contrast, the avian hippocampal area has no clearly visible layers. It consists of densely packed heterogeneous populations of neurons and it is not clearly distinguished from adjacent telencephalic structures. However, recent studies using detailed anatomical analysis of immunohistochemical and gene expression markers revealed the existence of layers in domestic chicks’ and adult chickens’ hippocampal formation (hippocampus proper and area parahippocampalis; Redies et al., 2001; Suárez et al., 2006; Abellán et al., 2014; Fujita et al., 2020, see Figure 1E; see Herold et al., 2019 for similar evidence in adult pigeons). This suggests that hippocampal layers are an ancestral trait at least among sauropsids. How these three layers of sauropsids compare to those in the mammalian hippocampus needs further clarifications.

Currently, there is no consensus on the divisions of the avian hippocampus and how they may correspond to the mammalian CA regions. Studies of the avian hippocampus vary widely in the number of proposed hippocampal subdivisions, in the boundaries of these divisions, and in their nomenclature (Figure 1). For instance, Craigie (1940), after examining the hippocampal formation of 16 different bird orders, divided this region into four longitudinal zones, each containing several layers and subdivisions. In pigeons, Karten and Hodos (1967) only distinguished parahippocampus (APH) and hippocampus proper (Hp). Krebs et al. (1991) suggested four subdivisions, while Erichsen et al. (1991) described seven subdivisions. Later studies in pigeons vary between five and seven subdivisions (Hough et al., 2002; Kahn et al., 2003; Herold et al., 2014), while other connectivity studies suggested nine subdivisions (Atoji and Wild, 2004, 2006; Atoji et al., 2016). Studies in zebra finches (Montagnese et al., 1996; Szekely, 1999), domestic chicks, and adult chickens (Suárez et al., 2006; Puelles et al., 2007; Gupta et al., 2012; Fujita et al., 2022) also vary in their proposed subdivisions and nomenclature.

The most debated issue is probably the position, or even the existence, of dentate gyrus within the avian hippocampus (Bingman and Muzio, 2017). Contrasting opinions have been proposed. Montagnese et al. (1996), Szekely and Krebs (1996), Szekely (1999), and Kahn et al. (2003) presumed the dentate gyrus to be located in the dorsomedial hippocampus of pigeons and zebra finches, respectively. On the contrary, Atoji and Wild (2004) located the dentate gyrus in the ventral part of the avian hippocampus, and the homologs of the cornu ammonis in the dorsal hippocampus. A more recent study of receptor distributions located the dentate gyrus in the ventrolateral part of the V-complex (the triangular part of the ventromedial region), while the dorsolateral portions of the pigeon hippocampal formation appeared to be more akin to the mammalian entorhinal cortex (Herold et al., 2014, see also lesion studies in Japanese quail Damphousse et al., 2022a). Developmental studies too locate the dentate gyrus in the ventral part of the hippocampal formation of domestic chicks and adult chickens (Suárez et al., 2006; Puelles et al., 2007; Gupta et al., 2012). One prominent feature of the mammalian dentate gyrus is that its medial layer contains granule cells, which project to the pyramidal cells of the CA3. Ramon y Cajal (1911) gave these axons the name “mossy fibers” because they appear like being covered in moss. Unfortunately, Timm’s staining, which reliably visualizes mossy fibers in mammals, does not allow to identify a comparable structure in birds (Faber et al., 1989; Montagnese et al., 1993, 1996; Herold et al., 2014). Timm’s staining reveals a relatively chaotic staining pattern in the hippocampal sections of domestic chicks (Faber et al., 1989), which could be more appropriately named “messy fibers” (if they are fibers at all). The pattern emerging from Timm’s staining, however, looks very similar in pigeons (Herold et al., 2014), domestic chicks (Faber et al., 1989), and zebra finches (Montagnese et al., 1996). This chaotic organization does not necessarily exclude the existence of “mossy fiber-like” circuits and dentate gyrus in birds’ hippocampus. However, the possibility that the dentate gyrus is an evolutionary innovation in mammals (Hevner, 2016), implying that there might be no homolog structure in birds, should also be seriously considered (Herold et al., 2014).

In summary, the existing evidence on the structure of avian hippocampus is remarkably contradictory. The avian and mammalian hippocampus has undergone divergent evolution over more than 300 million years. While the concept of homology is based on “sameness,” evolution introduces changes, making it difficult to define “sameness” (Faunes et al., 2015). Moreover, for the anatomical and functional comparisons of the hippocampus between such evolutionary distant species like birds and mammals, one should also consider the apparent differences and not only focus on the common traits (Striedter, 2015). Additional studies on a wider range of bird species are crucial to provide a more consistent view on the structure of the avian hippocampus. Comparisons with other sauropsids, complementing the usual mammal-avian dichotomy, could also be highly informative (Striedter, 2015).

## Is chick hippocampus involved in spatial orientation?

**Figure 2:**
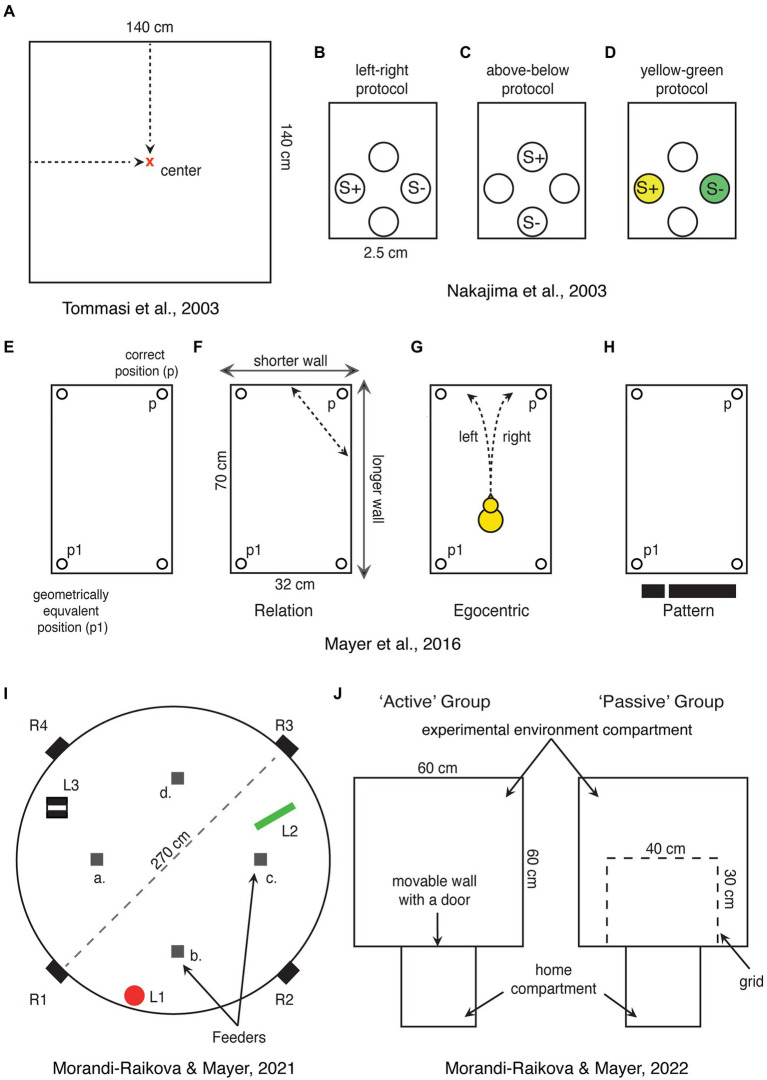
Schematic representations of spatial tasks that have been used for direct investigation of hippocampal involvement in domestic chicks. **(A)** Chicks are trained to find the center of a square-shaped arena, which can be encoded in relation to the walls or based on distance information. **(B–D)** View point dependent egocentric task, where chicks are trained to discriminate rewarded holes in one of the walls of the apparatus based on left–right or up–down positions or associated colors. S+ and S-represent rewarded and not rewarded positions, respectively. **(E)** Rectangular shaped arena used in the “Cheng” task, showing the correct position (p) and its geometrical equivalent (p1). **(F–H)** Alternative strategies that can be used to solve the “Cheng” task. In **(H)**, the black stripe represents the visual pattern provided by the short wall on the left and long wall on the right. **(I)** A large circular arena with free standing objects (L1—a red cylinder, L2—a green triangle, and L3—a striped box.), which can be used as a reference for finding the position of one rewarded feeder among four identical ones (a–d). In the alternative version of the task, the rewarded feeder can be marked by a distinct local cue. Chicks were released from four entrances (R1–R4) to disrupt egocentric orientation. **(J)** Experimental setup used to test the role of active exploration of a novel environment. Chicks were trained to enter the “experimental environment” through an open door. The active exploration group chicks could enter the “experimental environment” and actively explored it. Chicks of the “passive exploration” group could see the “experimental environment” through a grid, but not explore it actively.

Most spatial orientation studies in chicks were performed using a rectangular-shaped enclosure (Vallortigara et al., 2010; Tommasi et al., 2012). This classical paradigm was initially developed for rats (Cheng, 1986) and later applied to chicks (Vallortigara et al., 1990) and many other species (see below). In this task, the animals are trained to find a reward in one of the corners of a rectangular enclosure, using its shape as a reference for orientation. However, since a rectangular arena has two geometrically equivalent corners, animals choose equally often both the rewarded and the geometrically equivalent corner on the diagonally opposite side (Figure 2E). For this task, animals must be disoriented to disrupt their egocentric reference system. In a process called reorientation they recalibrate their disrupted egocentric representation in reference to the allocentric spatial information provided by the shape geometry of the arena.

Using this task, we found that the chicks hippocampus is involved in goal navigation in a rectangular enclosure (Mayer et al., 2016). Comparison of neural activity using the immediate early gene product c-Fos revealed strong activation of hippocampus in chicks trained to use geometrical information to locate the correct corners. On the contrary, chicks that were trained to rely on non-geometrical information (differently colored feeders in the corners of a square-shaped enclosure) showed higher activation of the medial striatum (Mayer et al., 2016). These results are in line with lesion studies in pigeons showing that birds’ hippocampus is crucial for reorientation in a rectangular arena (Vargas et al., 2004). However, what kind of information is processed by the hippocampus in this task?

Cheng (1986) originally proposed that animals possess a “geometric module,” which enables a representation of the geometrical shape of the environment (Cheng, 1986; Cheng et al., 2013). This has triggered a vast amount of comparative research, showing highly comparable behaviors across distant vertebrates in this task (e.g., rats: Cheng, 1986; monkeys: Gouteux et al., 2001; chicks: Vallortigara et al., 1990; pigeons: Vargas et al., 2004; fish: Sovrano et al., 2002; Lee et al., 2012; and humans: Hermer and Spelke, 1994). However, different strategies can be used to orient in this task, leading to a debate on the existence of the “geometrical module” (e.g., Sutton and Newcombe, 2014; Duval, 2019).

One strategy could be the use of relational information to encode the position of a goal in relation to the walls. Here, the long and short walls could be used as two distinct global landmarks and the position of the correct corner would be encoded in relation to them (Figure 2F). Such encoding of the position in relation to surrounding visual landmarks is comparable to the classical Morris water maze task with rats (Morris, 1981, 1984). In fact, in this task hippocampal lesions abolish positional encoding in relation to the distal visual cues outside the arena, but they do not affect orientation towards visible, local features of the goal in rats (Morris et al., 1982). Similar results have been obtained for zebra finches (Bischof et al., 2006; Watanabe et al., 2008; Mayer et al., 2010; Mayer and Bischof, 2012) and pigeons (Fremouw et al., 1997), demonstrating that also the avian hippocampus is involved in spatial orientation in relation to allocentric visual cues, but is not required for orientation towards local features (for a review, see Mayer et al., 2013).

Alternatively, in the rectangular arena of the “Cheng” task, it is also possible to encode the direction of the correct corner in relation to only one of the long walls. For instance, the animal could find the correct corner at the end of a long wall following it in a specific direction or it could estimate the angle (bearing) toward the correct corner using one of the long walls as a reference. While this strategy does allow to encode the precise position, it requires only knowledge of direction and if the used landmark (e.g., a long wall) is on the left or on the right side of itself in order to estimate the correct direction. Thus, the animal needs to rely on an egocentric reference system, which needs to be recalibrated in relation to the allocentric space. Egocentric information could also be used if the animal would reorient itself in parallel to the two long walls (i.e., facing a short wall) and then encode whether the correct corner is on the left or right side of itself (Figure 2G). This form of positional encoding is assumed to be a form of “egocentric” orientation, being view-point dependent (Vallortigara and Zanforlin, 1986; Vallortigara, 1996).

Interestingly hippocampus lesioned chicks are impaired in a view-point dependent “egocentric” task (Nakajima et al., 2003). Here the chicks were presented with four holes inside a wall where they could find food. They were then trained to discriminate left from right (Figure 2B), above from below (Figure 2C), or yellow from green (Figure 2D). The first two discriminations involve the egocentric reference system (the color discrimination was a control condition). Chicks with hippocampal lesions were delayed in acquisition of left–right and above-below discriminations. However, with further training, they were able to solve the task and to match the performance of control chicks (which implies identical recall performance between the two groups). As expected, the lesion did not impair the color discrimination task. Based on this study, it has been concluded that chicks’ hippocampus is involved in the acquisition of egocentric (non-geometric) information, but not in the recall of egocentric memories (Nakajima et al., 2003). Unfortunately, however, in this task it is not possible to exclude the use of geometric information. Chicks could have learned the absolute positions of the rewarded holes rather than egocentric information. For instance, the position of the baited feeder could have been encoded in relation to the other three feeders or to the walls, corners, the geometrical shape of the experimental chamber etc. It is thus possible that the effect of hippocampal lesions was due to the disruption of relational-allocentric encoding. Indeed, chicks with hippocampal lesions were able to learn the egocentric discrimination, even though at a slower rate, indicating that it is mainly processed outside hippocampus (hippocampus lesioned chicks might thus be able to rely only on egocentric information, while intact chicks might use also the relational information described above). It is worth mentioning that for successful navigation, the egocentric and allocentric reference systems need to be integrated. Animals need to know their position and orientation within an allocentric space. The role of avian hippocampus in the integration of egocentric and allocentric information needs to be further investigated.

Is it then “relational computing” what hippocampus does in general? In mammals spatial processing is considered a specific example of more general hippocampal functions: “relational computations” or relational learning (Cohen and Eichenbaum, 1993; Eichenbaum et al., 1999; Eichenbaum and Cohen, 2001; Day, 2003). Relational computations are also needed for encoding environmental geometry, which is a property that defines the position of a surface, line or point relative to the location of other objects or surfaces (Gallistel, 1990). The role of hippocampus would thus be to process geometrical relationships between visual cues. Because this property is processed by a distinct brain region, it can be seen as evidence supporting the idea of a “geometrical module.” In contrast, “non-geometric information” is a property that cannot be defined by relative position, such as the local features of the goal itself (Gallistel, 1990). In chicks, this information is processed outside the hippocampus, e.g., in the medial striatum (Mayer et al., 2016) or regions of the tectofugal visual pathway (Mayer et al., 2013; Clark and Colombo, 2022).

However, a true non-geometrical strategy has been also proposed for chicks’ reorientation in a rectangular arena (Pecchia and Vallortigara, 2010a). The short and the long wall can be seen as a two-dimensional (2D) image in a statistic visual field (Figure 2H). Thus, chicks could use visual pattern recognition or template matching between the currently perceived visual scene and a “snapshot” memory of the same (Pecchia and Vallortigara, 2010a). Template matching has been initially documented for orientation in invertebrates (Cartwright and Collett, 1983; Collett, 1992, 1995; Zeil, 1993; Wehner et al., 1996; Collett and Rees, 1997; Durier et al., 2003). The first evidence of visual template matching in birds comes from adult chickens (Dawkins and Woodington, 2000), and later pigeons (Pecchia et al., 2011) and hummingbirds (Pritchard and Healy, 2018; Pritchard et al., 2018). Whether this type of information is processed by birds’ hippocampus is not clear. Pattern discrimination is not expected to be hippocampus dependent in birds (Watanabe et al., 2008; Mayer et al., 2013; Clark and Colombo, 2022). However, visually responsive cells are actually present in pigeons’ hippocampus, but it has not been investigated yet what kind of visual information they process (Scarf et al., 2016). At a highly speculative level, one could hypothesize that hippocampus plays a role for binding a series of visual snapshots into “movie-like” memories. This would allow a representation of self-motion within an allocentric space. Interestingly, the hippocampus in pigeons receives a projection from the accessory optic system (Wylie et al., 1999), which processes optic flow and self-motion. However, studies targeting this issue in birds’ hippocampus still need to be performed.

Finally, other studies with chicks, questioned image-matching theories of reorientation in rectangular-shaped environments (Lee et al., 2012). Chicks successfully reoriented in a setup that contained three-dimensional (3D) borders, but they searched randomly when they had to reorient based on a flat (2D) rectangle depicted on the floor (Lee et al., 2012). It has been argued, that if chicks use view matching of 2D images, they should have been able to solve this task as well as the do in classical “Cheng” task. The study thus provides evidence that 3D borders play an important role for chick’s navigation. In a more recent study, we indeed found that chick’s hippocampus is sensitive to the boundaries of 3D layouts (Mayer et al., 2018). Chicks that were exposed to two enclosures of different geometric shapes activated the hippocampus to a greater degree compared to those exposed twice to the same environment. It is likely that exposure to environment of different shapes activated different populations of cells and thus a higher number of activated cells were found. On the contrary, two repeated exposures to the same environmental shape within a short period created an overlap in c-Fos activation of the same neuronal population, resulting in lower densities of c-Fos-immunoreactive cells. The results are in line with mammalian studies showing that different environments activate different populations of hippocampal cells (Vazdarjanova and Guzowski, 2004; Guzowski et al., 2005; Barry and Burgess, 2014). A recent study with Japanese quail (*Coturnix japonica*), using procedures that allow to distinguish immediate early gene expression following events occurring at different time points, confirmed these findings (Damphousse et al., 2022b). Overall, our study indicates that chicks’ hippocampus encodes boundary geometry and environmental shape (Mayer et al., 2018).

In summary, studies in chicks indicate that the avian hippocampus, like the mammalian one, encodes locations in relation to the global visual cues of the surroundings, but not based on local features (see also Colombo and Broadbent, 2000 for pigeons and Mayer et al., 2013 for zebra finches). Such an “allocentric” representation of spatial or geometrical relations between visual cues of an environment is in line with the classical idea that animals possess a ‘cognitive map’ (Tolman, 1948), which should be based on hippocampal computations (O’Keefe and Nadel, 1978). Further evidence of hippocampal involvement in chicks’ spatial orientation is provided in the following sections of this review.

## Are chicks’ hippocampal functions lateralized?

The first direct demonstration of the importance of the right hippocampus for processing spatial information in domestic chicks came from a hippocampal lesion study (Tommasi et al., 2003). Chicks were trained to find the center of a square-shaped enclosure (Figure 2A). Subjects that received a lesion to the left hippocampus performed like sham-operated and were able to find the center. However, lesions to the right hippocampus (or bilateral lesions) had a severe effect on chicks’ ability to find the center (Tommasi et al., 2003). Here it is important to consider the kind of information that the right hippocampus processed in this task. It has been discussed that the center of a squared enclosure can be found based either on absolute distances from the walls or on its relative position between the walls (Tommasi et al., 1997; Tommasi and Vallortigara, 2001). We argue that both orientation strategies are based on relational computations. Even in the first case, chicks need to estimate absolute distances from their viewpoints (they do not run to the walls to measure absolute distances to the goal location). However, viewpoint-based estimation of the distance between two objects depends on how far the animal is from them. Thus, the encoding mechanism needs to consider the animals’ own position relative to the objects the absolute distance between which needs to be estimated. Following this logic, both orientation strategies involve relational computations, for which the right hippocampus might play a predominant role in chicks.

To test this hypothesis, we used a task in which chicks had to orient in reference to free-standing objects within a large circular arena (Morandi-Raikova and Mayer, 2021). Crucially, the presence of the free-standing objects provided exclusively relational information for orientation (Figure 2I). We found that this form of spatial orientation is indeed predominantly processed by the right hippocampus (Morandi-Raikova and Mayer, 2021). Chicks that were trained to orient in reference to the free-standing objects had higher activation of the right hippocampus compared to their left and compared to chicks that oriented based on local features present at the rewarded location. The results highlight the right hippocampal role for retrieval of spatial relational memories in chicks (chicks were trained to find the rewarded location, before being tested, allowing us to observe activation during a “retrieval” task). Our study thus complements the hippocampal inactivation study by Tommasi et al. (2003). In this study, chicks were lesioned before training, demonstrating the involvement of right hippocampus in the learning phase. Together, these two studies suggest that the right hippocampus plays a dominant role for both acquisition and retrieval of spatial relational information in domestic chicks. However, they do not exclude that also the left hippocampus may play some role for spatial orientation in chicks. Indeed, in our studies, if chicks were tested either in a rectangular enclosure or exposed to novel shapes of the environments, we did not observe any right hippocampal advantage (Mayer et al., 2016, 2018; Morandi-Raikova and Mayer, 2020). Given that the left and right hippocampi are connected by the hippocampal commissure in birds, a functional linkage between the two sides is very likely (Atoji and Wild, 2006).

Other indirect evidence of a right hemispheric advantage for spatial orientation in chicks is based on behavioral studies, which used monocular occlusion. In studies using this technique, it is usually assumed, that information coming from each eye is mainly elaborated by the contralateral brain hemisphere (e.g., Rogers, 1996, 1997, 2008; Rosa-Salva et al., 2012; Rogers and Vallortigara, 2015). When it comes to spatial orientation, chicks orient better when using the left eye and thus presumably using the right hemisphere (Rashid and Andrew, 1989; Vallortigara et al., 1990; Tommasi et al., 2003; Vallortigara, 2004; Chiandetti et al., 2005). However, until recently, it has never been addressed to what degree monocular occlusion would affect neural activation in birds’ hippocampus.

To fill this gap, we exposed chicks to a novel environment, while either their left or right eye were occluded or in conditions of binocular vision (Morandi-Raikova and Mayer, 2020). We found, by measuring c-Fos expression, that the hippocampus showed increased activation only in chicks that explored the novel environment in condition of binocular vision. On the contrary, hippocampal activation in chicks that entered the novel environment either with their left or right eye occluded was not different from baseline. The results suggest that information from both eyes is needed to activate the hippocampus during spatial exploration. The simplest explanation is probably that in the monocular condition chicks are not very attentive to the global space. By seeing only half of the visual field, they were likely more focused on local features and did not recognize the spatial novelty, which was limited to the size and shape of a featureless environment (Morandi-Raikova and Mayer, 2020). Our results are in line with another study, where we tested chick’s orientation abilities in reference to three distinct objects inside a large circular arena (Morandi-Raikova et al., 2020). In this study too, information from both eyes was needed for successful spatial orientation. Chicks that could see from both eyes could find the position of a baited feeder in reference to the free-standing objects, which was not possible for chicks tested monocularly. On the contrary information from one eye (left or right) was sufficient for orientation based on local features, which was also the preferred strategy if spatial and local cues were put in conflict in this task, even for binocular chicks (Morandi-Raikova et al., 2020).

This represents a discrepancy with studies that reported successful spatial orientation in chicks using their left eye system (right eye occluded). This might be due to differences in incubation conditions of the eggs (Rashid and Andrew, 1989; Vallortigara et al., 1990; Tommasi et al., 2003; Vallortigara, 2004). In our studies, eggs were incubated in complete darkness, while in the spatial orientation studies mentioned above; chicks were obtained from local hatcheries, where eggs were likely exposed to light at least for brief periods. This is crucial, because exposure to light during incubation strongly influences the development of structural and behavioral asymmetries in domestic chicks (Rogers and Sink, 1988; Rogers and Bolden, 1991; Deng and Rogers, 1997, 2002; Rogers and Deng, 1999; Costalunga et al., 2021). Indeed, a study that compared light and dark incubated chicks on a spatial memory task, found that only light-incubated chicks with their left eye in use could solve the task (Chiandetti et al., 2005). On the contrary chicks from dark incubators were not able to use spatial information if they could see from one eye only (Chiandetti et al., 2005). The study indicates that embryonic light exposure crucially affects the development and lateralization of spatial functions in chicks. However, whether exploration of an environment under monocular vision condition would be enough to activate hippocampus in light-incubated chicks remains to be investigated.

In summary, our study (Morandi-Raikova and Mayer, 2021), together with the previous one by Tommasi et al. (2003), highlights the importance of the right hippocampus of domestic chicks in processing spatial information, which is in line with some mammalian literature (Maguire et al., 1998; Spiers et al., 2001; Iglói et al., 2010; for reviews, see Klur et al., 2009; Oleksiak et al., 2011). Without going so far as to speculate about the evolutionary ancestry of hippocampal lateralization in vertebrates, we would like to emphasize that a left hemispheric dominance has been repeatedly proposed for navigation in pigeons (Ulrich et al., 1999; Gagliardo et al., 2001, 2005; Prior et al., 2004; Bingman et al., 2006; Nardi and Bingman, 2007; Kahn et al., 2008; Mehlhorn et al., 2010; Jonckers et al., 2015; Griffiths et al., 2020). However, in pigeons, like in chicks, only few studies directly investigated hippocampal lateralization (Gagliardo et al., 2001, 2002, 2005; Nardi and Bingman, 2007; Kahn et al., 2008; Mehlhorn et al., 2010; Jonckers et al., 2015). Moreover, none of the above-mentioned studies are directly comparable to the only works that addressed this issue in chicks (Tommasi et al., 2003; Morandi-Raikova and Mayer, 2021). The only task used in both species is reorientation in a rectangular arena (“Cheng” task). In this task, hippocampal lesion in pigeons revealed a left hemispheric advantage (Nardi and Bingman, 2007), while we showed that in chicks both hippocampal sides were activated (Mayer et al., 2016). Unfortunately, so far hippocampal lesions in chicks have never been performed in this task, leaving the possibility that, in chicks too, the left hippocampus might be necessary also for this task, despite the activation results. It is still possible that the two sides of hippocampus in chicks and in pigeons may process different functions, possibly based on similar mechanisms. Future studies, using directly comparable tasks, need to investigate which specific information is processed by the left and by the right hippocampus and how the two hippocampi interplay to form spatial representations in chicks and in pigeons. This will allow to clarify if the difference between the two species is due to specific adaptations to rely on different strategies for orientation.

## Do chicks have place cells?

Many neurons in the rat and mouse hippocampus fire when the animal passes through a specific part of its environment. Place cells (O’Keefe and Dostrovsky, 1971; O’Keefe and Conway, 1978), head direction cells (Taube et al., 1990a,b), grid cells (Sargolini et al., 2006), border cells (Sargolini et al., 2006), speed cells (Kropff et al., 2015), and vector trace cells (Poulter et al., 2021) all contribute to the successful navigation in mammals (Moser et al., 2017; Poulter et al., 2018). However, so far only very few studies target this issue in any non-mammalian species.

Some pioneering works in pigeons (Bingman et al., 2003; Hough and Bingman, 2004, 2008; Siegel et al., 2005, 2006; Kahn et al., 2008) reported hippocampal cells with similar response properties to those of rats, which were however not so clearly defined as in rats (Bingman and Sharp, 2006; Hough, 2022). The first demonstration of clearly defined place cells in birds, came much more recently (Payne et al., 2021). In two different species of birds (tufted titmice, *Baeolophus bicolor* and zebra finches, *Taeniopygia guttata*), spatially responsive cells were recorded. These cells were found predominantly in the anterior hippocampus (their density decreased along the anterior–posterior axis, Payne et al., 2021). A similar gradient along the dorsoventral axis has been reported in rodents (Jung et al., 1994). Likewise, Agarwal et al. (2021) found place cells in the anterior hippocampus in freely flying barn owls. These studies thus support the idea that the anterior hippocampus is equivalent to the dorsal hippocampus in mammals (Figures 1M,N). Together these studies suggest that the neural mechanisms for spatial coding are evolutionary ancestral. Thus, also the hippocampus in chicks is expected to have place cells.

However, so far there are no studies showing place cells in chicks or adult chickens. Moreover, in a closely related galliform, the Japanese quail, the analysis of more than 2,000 hippocampal cells, did not reveal any place cells (Ben-Yishay et al., 2021). Instead about 12% of the cells were coding for head directions, similar to mammalian head directions cells (Taube and Muller, 1998). Based on these results, one could argue that place cells may exist only in some bird species and that spatial orientation abilities in quails may depend on other neural mechanisms (Damphousse et al., 2022b). This would point to a convergent evolution of place cells in mammals and some “advanced” bird species. However, we believe that place cells actually exist also in galliformes, but their density might be very low and making them difficult to find. Indeed, the hippocampus of zebra finches (non-food hoarding specie) exhibited less place selective cells compared to the food-hoarding titmice (Payne et al., 2021). Even fewer may exist in galliformes, such as quails and chickens, which retained more ancestral traits compared to neoaves (Prum et al., 2015). This hypothesis is supported by our most recent paper (Morandi-Raikova and Mayer, 2022). In our study, chicks that could actively explore an environment had a higher c-Fos expression in the anterior hippocampus and in the dorsolateral parts of the intermediate hippocampus, compared to those that passively observed the same environment from a restricted area (Figure 2J). As we predicted, physical movement across different locations contributed to hippocampal activation. Indeed, the number of visited sectors positively correlated with the activation of the anterior hippocampus. Even though this study represents a rather indirect measurement, the findings suggest that, in birds like in mammals, the increase in hippocampal c-Fos expression, during exploration of an environment reflects the increased firing rates of spatially coding neurons. We believe that the more locations (“place fields”) were visited, the larger the number of different spatially coding cells was activated (Morandi-Raikova and Mayer, 2022).

In summary electrophysiological confirmation studies with domestic chicks are urgently needed at this point. Our study (Morandi-Raikova and Mayer, 2022) together with studies in other birds (Agarwal et al., 2021; Payne et al., 2021) indicate that future studies in chicks should focus in on the anterior hippocampus, which increased the density of c-Fos immunoreactive cells after active exploration of a novel environment. Moreover, it is important to investigate if also other spatially coding cells (e.g., head direction cells, border cells, or grid cells) are existent in chicks’ hippocampi.

## Conclusion

To sum up, all the questions tackled in the subsections of this review can be answered in the affirmative. Chicks have a hippocampus homologue brain region, which processes spatial information, whose functions are lateralized and likely based on spatially coding cells. The use of domestic chicks as a model complements the existing literature on avian hippocampus, which is mainly based on studies on adult animals. This precocial model species thus allows to study the development of spatial coding in the avian hippocampus. The studies presented in this review provide the starting point for this research field. Moreover, Galliformes like domestic chickens and quails present some more ancestral traits compared to neoaves, and can be thus informative for the understanding of the evolution of hippocampal spatial functions.

However, although a number of studies have targeted the structure and function of the avian hippocampus, the resulting information leaves unanswered many fundamental questions, especially from a comparative perspective. Possibly, the most controversial point for a comparative analysis is the obvious anatomical dissimilarity of the avian and the mammalian hippocampal structure, despite their functional similarities. Moreover, for a full comparison between domestic chickens and other species, it needs to be investigated first how far spatial functions reported for young chicks hippocampi would remain in adulthood. This should be done using directly comparable tasks and training procedures. For instance some interesting effects discovered in pigeons (“big box-little box” effect as well as the role of hippocampus for incidental processing of information, Johnston et al., 2021) have never been investigated in chicks or adult chicken. In particular this direct comparative approach could be important for our interpretations of discrepancies in lateralization and hippocampal subdivisions across avian species. Furthermore, electrophysiological confirmation studies with domestic chicks are needed to investigate how the chicken anterior hippocampus encodes locations, and if it contains anything comparable to place and head directions cells reported in other birds. If that would be the case, we could study the development of hippocampal spatial representations starting from naïve animals with well controlled pre-hatching experience in comparison to animals raised in controlled conditions. Finally, it is important to stress that this review focuses only on the spatial functions of chicks’ hippocampus. For a complete understanding of the evolution of this crucial structure, non-spatial hippocampal functions need also to be included in the picture. Studies with different bird species are also needed to extend the existing evidence and to provide a unified view on the structure and function of avian hippocampus. Structural and functional investigations of hippocampus homologues in other sauropsids may also be informative on the evolution of this structure.

## Author contributions

UM conceptualized the paper. AM-R and UM jointly wrote the manuscript. All authors contributed to the article and approved the submitted version.

## Funding

UM and AM-R are funded by the University of Trento.

## Conflict of interest

The authors declare that the research was conducted in the absence of any commercial or financial relationships that could be construed as a potential conflict of interest.

## Publisher’s note

All claims expressed in this article are solely those of the authors and do not necessarily represent those of their affiliated organizations, or those of the publisher, the editors and the reviewers. Any product that may be evaluated in this article, or claim that may be made by its manufacturer, is not guaranteed or endorsed by the publisher.
